# The Putative Role of *TIM-3* Variants in Polyendocrine Autoimmunity: Insights from a WES Investigation

**DOI:** 10.3390/ijms252010994

**Published:** 2024-10-12

**Authors:** Andrea Ariolli, Emanuele Agolini, Tommaso Mazza, Francesco Petrizzelli, Stefania Petrini, Valentina D’Oria, Annamaria Cudini, Caterina Nardella, Vanessa Pesce, Donatella Comparcola, Marco Cappa, Alessandra Fierabracci

**Affiliations:** 1 Bambino Gesù Children’s Hospital, Istituto di Ricovero e Cura a Carattere Scientifico (IRCCS), 00146 Rome, Italy; andrea.ariolli@opbg.net (A.A.); tommaso.mazza@opbg.net (T.M.); annamaria.cudini@opbg.net (A.C.); caterina.nardella@opbg.net (C.N.); vanessapesce2018@gmail.com (V.P.); donatella.comparcola@opbg.net (D.C.); 2 Laboratory of Medical Genetics, Translational Cytogenomics Research Unit, Bambino Gesù Children’s Hospital, Istituto di Ricovero e Cura a Carattere Scientifico (IRCCS), 00146 Rome, Italy; emanuele.agolini@opbg.net; 3 Laboratory of Bioinformatics, Casa Sollievo della Sofferenza, Istituto di Ricovero e Cura a Carattere Scientifico (IRCCS), 70013 San Giovanni Rotondo, Foggia, Italy; f.petrizzelli@css-mendel.it; 4 Confocal Microscopy Core Facility, Bambino Gesù Children’s Hospital, Istituto di Ricovero e Cura a Carattere Scientifico (IRCCS), 00146 Rome, Italy; stefania.petrini@opbg.net (S.P.); valentina.doria@opbg.net (V.D.); 5 Research Unit Innovative Therapies for Endocrinopathies, Bambino Gesù Children’s Hospital, Istituto di Ricovero e Cura a Carattere Scientifico (IRCCS), 00146 Rome, Italy; marco.cappa@opbg.net

**Keywords:** autoimmune polyendocrine syndrome, etiopathogenesis, WES, SNP, TIM-3

## Abstract

Autoimmune polyglandular syndrome (APS) comprises a complex association of autoimmune pathological conditions. APS Type 1 originates from loss-of-function mutations in the autoimmune regulator (*AIRE*) gene. APS2, APS3 and APS4 are linked to specific HLA alleles within the major histocompatibility complex, with single-nucleotide polymorphisms (SNPs) in non-HLA genes also contributing to disease. In general, variability in the *AIRE* locus and the presence of heterozygous loss-of-function mutations can impact self-antigen presentation in the thymus. In this study, whole-exome sequencing (WES) was performed on a sixteen-year-old female APS3A/B patient to investigate the genetic basis of her complex phenotype. The analysis identified two variants (p.Arg111Trp and p.Thr101Ile) of the hepatitis A virus cell receptor 2 gene *(HAVCR2*) encoding for the TIM-3 (T cell immunoglobulin and mucin domain 3) protein. These variants were predicted, through in silico analysis, to impact protein structure and stability, potentially influencing the patient’s autoimmune phenotype. While confocal microscopy analysis revealed no alteration in TIM-3 fluorescence intensity between the PBMCs isolated from the patient and those of a healthy donor, RT-qPCR showed reduced TIM-3 expression in the patient’s unfractionated PBMCs. A screening conducted on a cohort of thirty APS patients indicated that the p.Thr101Ile and p.Arg111Trp mutations were unique to the proband. This study opens the pathway for the search of *TIM-3* variants possibly linked to complex autoimmune phenotypes, highlighting the potential of novel variant discovery in contributing to APS classification and diagnosis.

## 1. Introduction

Autoimmune polyglandular syndrome (APS) includes four main categories of complex associations of autoimmune diseases [1,2,3]. Autoimmune polyglandular syndrome Type 1 (APS1), namely Autoimmune–Polyendocrinopathy-Candidiasis-Ectodermal dystrophy syndrome (APECED, OMIM #240300, ORPHA 3453), is determined by loss-of-function mutations in the autoimmune regulator (*AIRE*) gene; the presence of at least two of the following disorders—chronic mucocutaneous candidiasis (CMC), chronic hypoparathyroidism (HP) and primary adrenal insufficiency (Addison’s disease, AD)—confirms clinical diagnosis [4]. The incidence of APS2 to 4 in published cohorts is estimated to be between 1.4 and 4.5 per 100,000 inhabitants [5].

In more detail, APS2 (Schmidt’s syndrome, OMIM #269200, ORPHA 3143) includes the combination of autoimmune thyroid disease (AITD), Type 1 diabetes mellitus (T1D) and AD. APS3 refers to the association between AITD and one or more autoimmune diseases (AIDs) excluding AD. Since AITD is one of the most frequent autoimmune diseases, APS3 is the most prevalent APS worldwide. APS3 (ORPHA 227982) includes subgroups APS3A-APS3D based on the associated AIDs: APS3A (AITD and endocrinopathies excluding AD), APS3B (AITD with gastrointestinal, hepatic or pancreatic autoimmunity), APS3C (referring to the coexistence of AITD with skin, neurological and hematological diseases), and APS3D (AITD presenting with autoimmune rheumatological, cardiac and vascular diseases) [1,2,6,7]. APS4 (ORPHA 227990) includes any other AID combination that cannot be diagnosed as APS1, APS2 or APS3 [1,2].

APS2, APS3 and APS4 are strictly associated with peculiar HLA alleles of the major histocompatibility complex (MHC) located on chromosome 6 [5]. Further, their pathogenesis is contributed to by the SNPs of several susceptibility non-HLA genes [3,5], including the *PTPN22* (protein tyrosine phosphatase non-receptor type 22) C1858T variant encoding for the R620W (rs2476601) Lyp frequently discovered in patients with T1D, AITD, AD and APS2 syndrome [8,9]. Other gene polymorphisms associated with APS are detected in the *CTLA4* (cytotoxic T lymphocyte antigen 4) gene [10]; the *IL2ra* gene encoding for IL2Ra (interleukin 2 receptor alpha) (CD25) [11]; the *TNFα* (tumor necrosis factor alpha) gene [12,13]; the *FOXP3* (forkhead box P3) gene, which controls T regulatory cell (Treg) development and function [14]; and MHC class I chain-related gene A (*MICA*) [15,16]. Further, T1D susceptibility is influenced by the variable number of tandem repeats (VNTR) of the insulin gene [17,18]. It is generally recognized that *AIRE* locus variability and the presence of heterozygous loss-of-function *AIRE* mutations can affect the presentation of self-antigens at the thymus level [19]. *AIRE* variants have been detected in organ-specific autoimmune conditions [20,21,22].

In future studies, whole-exome sequencing (WES) may be useful in improving our understanding of the genetic causes of APS in different cohorts of patients, leading to the identification of undiscovered SNPs in non-HLA genes [23]. This could also contribute to the classification of APS and prediction strategies of the evolution of its clinical autoimmune characteristics within each category.

In the light of the foregoing, in this manuscript, WES investigation was conducted on the DNA of a female patient affected by APS3A/3B in order to explore the underlying genetic pathogenetic mechanism responsible for the complex phenotype. This study led to the identification of two compound heterozygous missense variants of the hepatitis A virus cell receptor 2 gene (*HAVCR2*) [OMIM #606652] encoding for the transmembrane protein TIM-3 (T cell immunoglobulin and mucin domain 3) [24]. The putative pathogenetic significance in the autoimmunity of the identified variants was unraveled in functional studies by confocal microscopy analysis, RT-qPCR and the screening of a cohort of polyendocrine patients.

## 2. Results

### 2.1. Identification and Structural Characterization of TIM-3 Variants

**Figure 1:**
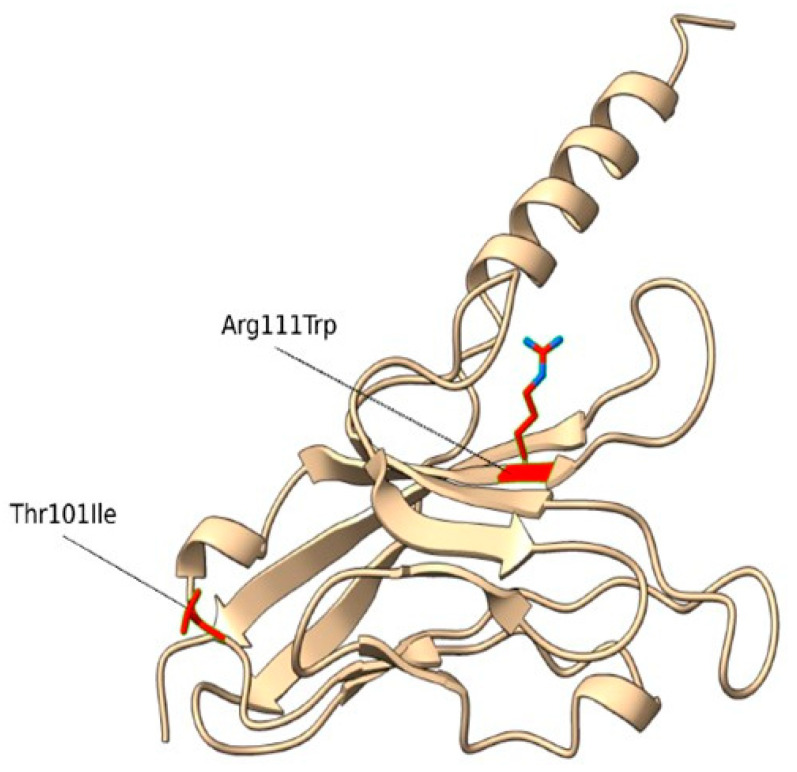
Homology model of the human TIM-3 protein. The Arg residue (site of the p.Arg111Trp mutation) and Thr (site of the p.Thr101Ile mutation) are indicated by red sticks.

The WES analysis identified 2 compound heterozygous missense variants in the *TIM-3* (*HAVCR2*) gene, c.331C>T (p.Arg111Trp) and c.302C>T (p.Thr101Ile) (Figure 1, Table 1).

Phases were determined directly using data from sequencing reads. The variant p.Arg111Trp (rs145478313) was shown to have an effect in sporadic subcutaneous panniculitis-like T cell lymphoma [25], theoretically affecting membrane phosphatidylserine [26]. The variant p.Thr101Ile (rs147827860) was reported by Tromp and Gillissen [27], and they showed that it abrogates TIM-3 expression.

**Figure 2:**
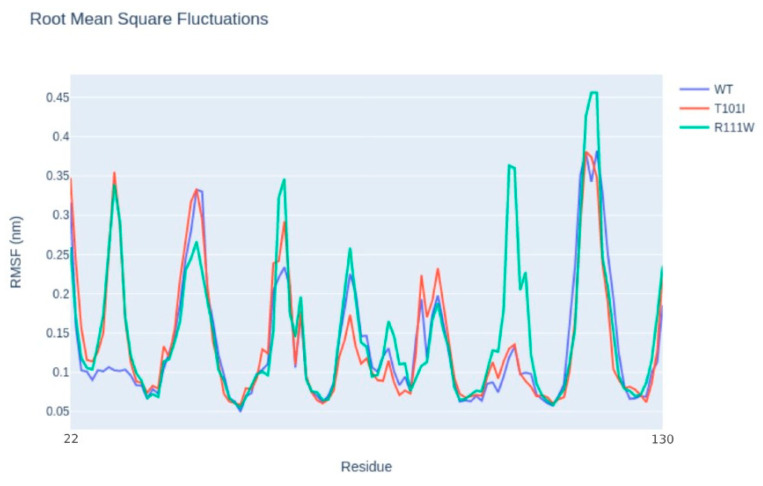
Molecular dynamics simulations. Snapshots of RMSF (root mean square fluctuation) values of molecular dynamics simulations of the R111W and the T101I variants and the wild-type TIM-3 protein are shown.

Then, we conducted a structural characterization of the two variants to estimate their impact on protein structure, stability and dynamics. According to Missense3D, p.Arg111Trp substitution determined the breakage of fundamental interactions in a buried region of the TIM-3 protein while no structural impact was detected for the p.Thr101Ile variant. Similarly, the difference in free energy, ΔΔG, resulted in a destabilizing effect on the p.Arg111Trp (ΔΔG(p.Arg111Trp) = 6.38599 Kcal/mol) structure, while no impact on protein stability was found for the p.Thr101Ile variant (ΔΔG(p.Thr101Ile) = −0.52554 Kcal/mol). Finally, molecular dynamics simulation-based evaluation was employed to determine the impact of the variants on the protein motions. In detail, the RMSF profiles (Figure 2), which provide a quantitative measure of the residue fluctuation during the simulation, show different fluctuation patterns, suggesting that the variants may affect the dynamic behavior of the molecule.

In particular, p.Arg111Trp shows multiple distinct peaks compared with the wild type, especially around residues 100 to 130, where the variant introduces additional flexibility or disorder in that specific region of the molecule. On the other hand, p.Thr101Ile shows a very similar fluctuation pattern to the wild type, with a distinct peak only at the region made by residues 22 to 30. Finally, in comparing the molecular interactions such as hydrogen bonds and van der Waals interactions, the high flexibility observed with the p.Arg111Trp mutant is caused by the loss of a fundamental hydrogen bond between the mutant residue and asparagine 120, with a clear impact on the stability of the entire protein. On the contrary, the p.Thr101Ile contact network resembles the wild-type profile except for the increased interaction frequencies between the mutant residue and arginine 81, which could have resulted in the increased flexibility observed in the RMSF profile.

### 2.2. Evaluation of TIM-3 Expression on PBMCs

**Figure 3:**
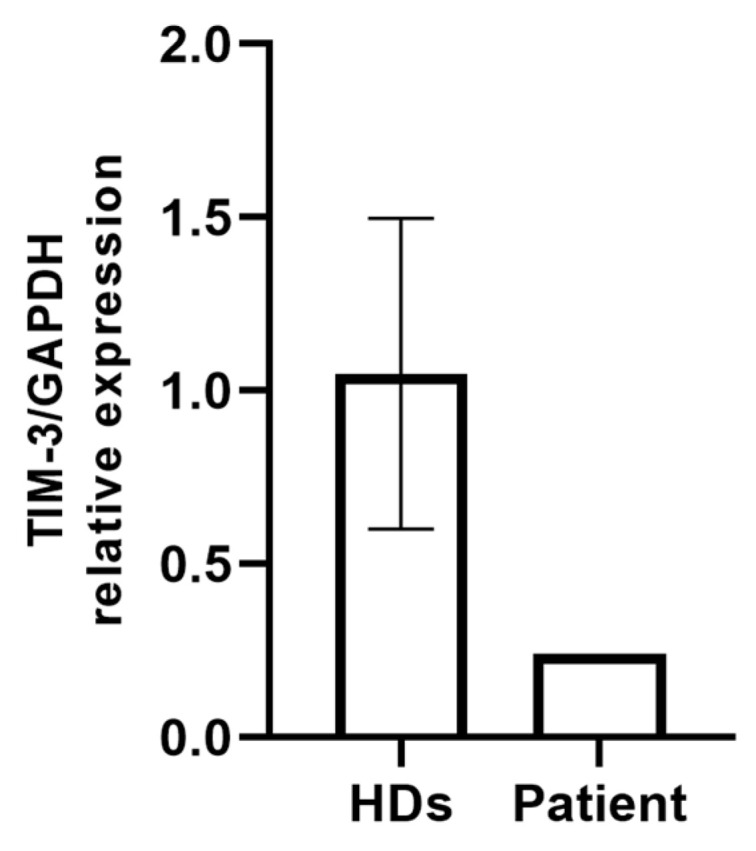
RT-qPCR analysis of TIM3 expression in PBMCs isolated from two healthy donors and the patient carrying p.Arg111Trp and p.Thr101Ile variants. Gene expression levels were normalized to GAPDH.

LSCM analysis of PBMC preparations from the patient and two healthy controls did not show differences in the TIM-3 localization and intensity (Supplementary Figure S1). However, RT-qPCR analysis revealed lower TIM-3 mRNA levels in the patient cells compared with the healthy donor-derived PBMCs (Figure 3).

### 2.3. Exon 2 TIM-3 Sequencing in APS Patients’ DNAs

In a cohort of 30 polyendocrine patients, no patient harbored exon 2 *TIM-3* c.331C>T (p.Arg111Trp), and the c.302C>T (p.Thr101Ile) variants reported in the proband showed that the disease association could be influenced by the eventual presence of *AIRE* SNPs (5 patients presenting the heterozygous intronic IVS9+6G>A polymorphism and 3 patients harboring the S278R (c.834C>G variant)) [28], *AIRE* promoter SNPs (present in 12 patients) [3] and the C1858T *PTPN22* variant (present in 8 patients) [8] (Supplementary Table S1).

## 3. Discussion

With the aim to identify the molecular etiology of polyendocrine autoimmune syndrome, WES studies were conducted on the DNA of an APS3 patient, leading to the identification of the compound heterozygous c.331C>T (p.Arg111Trp)/c.302C>T (p.Thr101Ile) variants of *TIM-3*. Molecular dynamic profiling and functional studies led to the hypothesis that mutations could affect the patient’s autoimmune phenotype by reducing protein stability. In particular, evidence is provided that p.Arg111Trp affects TIM-3 structure and stability and both mutants influence protein dynamics.

TIM-3 is a type I transmembrane protein located on chromosome 5q33.2 (protein length: 302 amino acids) and composed of four parts: variable immunoglobulin domains (IgVs), mucin domains, transmembrane regions and intracellular stems [29]. Four TIM-3-related proteins have so far been identified as ligands for TIM-3, interacting with its IgV domain to mediate signal transduction; these include Gal-9, important for maintaining cell homeostasis and inflammation, and phosphatidylserine (PtdSer), whose interaction with TIM-3 promotes the phagocytosis of apoptotic bodies and enhances the cross-antigen presentation of dendritic cells [30,31]. Other ligands are the DNA-binding protein HMGB1 [32] and carcinoembryonic antigen cell adhesion molecule 1 (Ceacam 1) [33].

TIM-3 was initially reported to be expressed on the surfaces of Th1, Th17, innate immune cells such as monocytes and macrophages, NK and dendritic cells and cancer stem cells [24,29,34,35,36,37]. The TIM-3 protein and its ligands can cause peripheral immune tolerance, and blocking TIM-3 can eliminate the development of tolerance in Th1 lymphocytes [38].

In recent years, although the mechanisms of its complex regulatory functions have not been fully elucidated, the putative immune checkpoint TIM-3 has received increasing attention in reference to chronic infection diseases to mediate an anti-tumor immune response and affect the adaptive immune response in autoimmune diseases [29]. This evidence has envisaged that TIM-3 could serve as a potential target for breakthrough immunotherapy [29]. Several single-nucleotide polymorphisms (SNPs) were identified in the *TIM-3* gene underlying rheumatoid arthritis (RA) susceptibility [39]. Furthermore, the SNPs of the *TIM-3* promoter region were postulated to affect susceptibility to multiple sclerosis [40]. The expression of TIM-3 was correlated with disease activities in patients with active systemic lupus erythematosus (SLE) [41]. Further, TIM-3 is an important negative regulator of T cell function in patients affected by multiple sclerosis (MS) and may cause autoreactive T cells and pathogenic T cells to escape negative regulation [42]. TIM-3 dysregulation in Th1 cells was also found in patients with Crohn’s disease [43] and correlated with imbalanced CD4 helper T cell function in ulcerative colitis [44]. Reduced expression of TIM-3 was reported in T1D patients and may affect the Th1/Th2 balance, thus putatively participating in disease progression [45]. TIM-3 mainly inhibits Th1 cells, thus acting as an anti-inflammatory molecule, while TIM-1 regulates T cell activation, being mainly expressed on Th2 cells. The ratio of TIM-3 to TIM-1 was decreased in the T1D patients, most notably in the defective islet function group. Further, the TIM-3/TIM-1 ratios in the different T cell subsets were increased in the remission phase of the disease, indicating an imbalance between pro- and anti-inflammatory activities. Evidence also indicates that the molecular mechanism of TIM-3 is also involved in human allergic reactions [29] and the induction of respiratory tolerance in experimental asthma [46].

In further unraveling the putative effect of the compound heterozygous c.331C>T/c.302C>T genotype of *TIM-3* in the patient analyzed in the present investigation, we could not find evidence of qualitative or quantitative differences with confocal microscopy examination between the healthy control and patient-derived PBMCs. Nevertheless, an in-depth evaluation by RT-qPCR evidenced a reduced expression of TIM-3 in the patient’s unfractionated PBMCs compared with in the two healthy controls, further supporting the hypothesis of the putative functional effect on protein stability and immunological function. This apparent discrepancy may arise from several factors. Notably, mRNA levels do not always correlate directly with protein amounts. As TIM-3 is expressed on multiple immune cell types, changes in mRNA levels could reflect shifts in cell population composition (as would be expected in relation to immune disease pathogenic mechanisms) rather than altered TIM-3 expression in individual cells, such as an increased proportion of cells expressing lower transcript levels. Further, the impact of the mutations may differ across distinct immunotypes, thus requiring more in-depth molecular analysis.

The identified *TIM-3* exon 2 genetic variants were not present in the DNAs of 30 additional polyendocrine patients. Nevertheless, the result of the present investigation opens the pathway to discovering novel *TIM-3* variants that could underlie the pathogenesis of a complex autoimmune phenotype in APS patients and identify a specific clustering of association with peculiar clinical manifestations, thus contributing to APS classification and diagnosis.

## 4. Materials and Methods

### 4.1. Patients

#### 4.1.1. Case History

A 16.4-year-old girl from the central region of Italy, affected by patent (Botallo) ductus arteriosus [47], was admitted to Children’s Hospital Bambino Gesù at the age of 1.8 years with symptoms of polyuria, polydipsia, hyperglycemia and severe ketoacidosis. A diagnosis of T1D was ascertained, and the patient started insulin substitutive treatment. The patient also presented IgA deficit. At the age of 3.5 years, the patient was diagnosed to be affected by celiac disease. At the age of 5 years, the patient underwent surgery to percutaneously close the patent ductus arteriosus with an Amplatzer device. At the age of 12 years, metformin treatment was added to insulin administration. At the age of 13 years, the patient had an epileptic crisis and preclinical autoimmune thyroiditis was diagnosed, and at 13.3 years old, hypertransaminasemia was detected with increased markers of hepatic cytolysis and elevated levels of IgG, anti-LKM (liver-kidney-microsomal) autoantibodies (Ab) and ASMAs (Table 2).

At a histopathological examination, chronic autoimmune hepatitis with partially disturbed architecture was confirmed due to the presence of portoportal arched septa. In the portal spaces, found expanded due to fibrosis and with an irregular profile, a dense lymphomonocytic CD3+ CD20+ inflammatory infiltrate was observed with an advancing MUM1 (multiple myeloma oncogene-1)-positive front rich in plasma cells exceeding the limiting lamina (interface hepatitis). A similar but smaller infiltrate was also described in the lobular site with particle necrosis. The patient underwent cyclosporine and azathioprine treatment, leading to macrocytosis.

At the age of 16.2 years, D hypovitaminosis, leukopenia and neutropenia were found. For the cohort of autoimmune clinical manifestations, according to the updated APS classification [2], the patient could be diagnosed as APS3A/B (Table 2).

#### 4.1.2. Study Population

Thirty patients affected by APS, i.e., variable association of organ- and non-organ-specific autoimmune disorders (12 males, 18 females with age ranges at presentation between 0.9 and 19.6 years old), were recruited at Bambino Gesù Children’s Hospital (OPBG) in Rome for the screening of the exon 2 TIM-3 mutations identified in the proband (vide infra) (Supplementary Table S1).

According to the current criteria for the classification of APS [1,2,48,49], 1 patient was affected by APS2 (patient n° 22), 17 patients were affected by APS3A (patients n° 1, 2, 7–9, 11–13, 15, 19, 21, 24–29), 8 patients were affected by APS3B (patients n° 1, 8, 11, 13, 19, 22, 23, 30), 5 patients were affected by APS3C (patient n° 2, 6, 18, 21, 30), one patient was affected by APS3D (patient n° 6) and 10 patients were affected by APS4 (patients n° 3–5, 7, 10, 14, 16, 17, 20, 27).

Informed consent was obtained from all those who took part in the present study, including the proband, in accordance with the Declaration of Helsinki. The investigation was approved by the local Institutional Review Board (IRB) of OPBG, which regulates human sample usage for experimental studies (study protocol no. 1385_OPBG_2017).

### 4.2. Autoantibody Screening

The patients’ sera were assayed for T1D-related autoantibodies (Ab), i.e., glutamic acid decarboxylase isoform 65 (GADAb), tyrosine phosphatase-related islet antigen 2 (IA2Ab), insulin Ab (IAAs) and zinc transporter 8 Ab (ZnT8Ab), by an enzyme-linked immunosorbent assay (ELISA); for AD, i.e., adrenal cortex antibodies (ACAs) and 21-hydroxylase Ab (21-OHAb), by an ELISA; for AITD-related Ab, i.e., thyrotropin (TSH)-receptor Ab (TRAb), by an immunoassay (Immulite TSI; Siemens Healthcare, Tarrytown, NY, USA); and thyroglobulin (TgAb) and thyroperoxidase Ab (TPOAb) via an electrochemiluminescence immunoassay (ECLIA) (Siemens, Erlangen, Germany). Celiac-disease-related Ab were screened by chemiluminescence (ADVIA Centaur analyzer; Siemens Healthcare, Germany), i.e., anti-transglutaminase-IgA Ab (TRGAb), and deaminated gliadin-IgG Ab (DGP-IgGAb) by EliA. The sera were also assayed for autoimmune hepatic diseases, i.e., anti-liver kidney microsomal Ab (LKMAb), smooth muscle Ab (SMAs), liver cytosol type 1 Ab (LC1Ab) and soluble liver antigen/liver pancreas Ab (SLA/LPIgG); stomach-related Ab, i.e., parietal cell Ab (APCAs), were measured by IFL, and intrinsic factor Ab (IFIAb) were tested by an ELISA. Anti-SP100 Ab and anti-glycoprotein GP210 were tested by immuno-dot and anti-Saccharomyces cerevisiae Ab by an ELISA. Non-organ-specific Ab, i.e., nuclear Ab (ANAs), extractable nuclear antigens (ENAs) (ELiA, Thermo Fisher, Waltham, MA, USA), neutrophil cytoplasmic Ab (ANCAs), double-stranded DNA Ab (dsDNAAb), SCL-70 antigen Ab (SCL-70Ab), reticulin Ab (ARAs), mitochondrial Ab (AMAs), ribosomal Ab (RAb), phospholipid Ab or anti-cardiolipin Ab (CAb), beta2glycoprotein I-IgG or IgM Ab (β2GP-1IgGAb), anti-beta2glycoprotein I-IgM (β2GP-1IgMAb), dense fine speckled 70 Ab (DSF70Ab) (ELiA) and cyclic citrullinated peptide Ab (CAb) (EliA), were also tested.

### 4.3. Molecular Studies

Genomic leukocyte DNA was extracted from the patient’s whole blood by the QIAmp DNA Blood Mini Kit (Qiagen, Hilden, Germany) according to the manufacturer’s guidelines.

#### 4.3.1. *AIRE* Gene Screening

All 14 exons and intronic regions of the *AIRE* gene were sequenced according to already described protocols in the DNAs of recruited patients [50]. The *AIRE* promoter SNPs were also identified as previously reported [3].

#### 4.3.2. Screening for the Presence of C1858T *PTPN22*

Detection of the C1858T variant in the *PTPN22* gene was carried out by PCR with specific primers for exon 14 of the *PTPN22* gene (GenBank ID: 26191): forward 5′-GATAATGTTGCTTCAACGGAATTT-3′ and reverse 5′-CCTCAAACTCAAGGCTCACAC-3′. The amplification lasted thirty-five cycles, generating PCR products of 318bp that were purified using the NucleoSpin Gel and PCR Clean-Up kit (Macherey-Nagel, Düren, NRW, Germany). PCR sequencing was carried out with the BigDye Terminator v.3.1 Cycle sequencing protocol (Life Technologies, Applied Biosystems, Paisley, Scotland, UK) on a 3500 Genetic Analyzer (Applied Biosystems) as reported [8,51].

#### 4.3.3. Whole-Exome Sequencing

After the obtaining of informed consent of the patients for the genetic testing, patient genomic DNA was extracted as described above. Library preparation was carried out by using the Twist Human Comprehensive Exome enrichment kit, according to the manufacturer’s protocol (Twist Bioscience, South San Francisco, CA, USA), and sequenced on a NovaSeq 6000 (Illumina, Inc., San Diego, CA, USA) platform. The BaseSpace pipeline (Illumina, https://basespace.illumina.com/, accessed on 21 December 2023) and Geneyx Analysis (an AI-driven NGS Analysis platform), formerly known as TGx Gene Cards, were used for the variant calling and annotating variants, respectively. Sequencing data were aligned to the hg19 human reference genome. The variants were analyzed in silico by using Combined Annotation-Dependent Depletion (CADD) V.1.3 [52], Sorting Intolerant from Tolerant (SIFT) [53], Polymorphism Phenotyping v2 (PolyPhen-2) [54] and Mutation Taster (v2021) [55] for the prediction of deleterious, non-synonymous variants in human diseases. In summary, the pathogenicity of each variant was evaluated by gathering evidence from the above sources, including population data, computational and predictive data and segregation data, in accordance with the guidelines of the American College of Medical Genetics and Genomics (ACMG) [56]. Variants were examined for coverage and Qscores (minimum threshold of 30) and visualized by the Integrative Genomics Viewer (IGV).

#### 4.3.4. Structural Modeling and Simulation

A high-resolution crystal structure of the IgV-like domain (from residue 22 to residue 130) of the TIM-3 protein was retrieved from the RCSB Protein Data Bank (PBD ID: 7M3Z). Then, a comprehensive structural evaluation was performed to characterize the impact of the two missense variants, p.Arg111Trp and p.Thr101Ile, on the TIM-3 protein structure. In detail, the Missense 3D web tool [57] was employed to predict the structural changes introduced by the mutations. Then, the stability of both mutant structures was assessed through the BuildModel function implemented in FoldX v.5.0 [58], run using standard parameters.

Finally, we employed advanced molecular dynamics (MD) simulation techniques [59] to further describe the eventual variant perturbations on the protein dynamics, following the protocol described by Cocciadiferro and Mazza [60] and resorting to gaming-enabled GPU graphic cards [61]. In brief, the wild-type structure was mutated in silico to introduce the two missense variants, and each system was embedded into a simulation box, extending up to 12 Å, filled with TIP3P water molecules and Na^+^ and Cl^−^ counter-ions, using the web-based tool CHARMM-GUI Enhanced Sampler [62]. Then, each system was firstly subjected to energy minimization to refine potential clashes, gradually heated to 300 K and equilibrated for 500 ps and finally simulated using a standard “dual-boost” Gaussian accelerated MD (GaMD) simulation protocol, which included a preparatory 2 ns run to collect potential statistic, an 8 ns “equilibration run” and a final production run of 200 ns. Each MD simulation was then analyzed using GROMACS v.2018, measuring the root mean square deviation (RMSD) and the root mean square fluctuation (RMSF), respectively, to assess the average distance and deviation over time between the positions of the Cα atomic coordinates of each residue and those of the starting structure, while all fundamental interactions and their residence times during each trajectory were computed using the GetContacts (https://getcontacts.github.io, accessed on 1 June 2024) tool.

#### 4.3.5. Screening for the Presence of Exon 2 *TIM-3* Variants

The presence of the identified SNPs of the T cell immunoglobulin and mucin domain-containing protein 3 (TIM-3, GenBank ID: 84868) gene was analyzed in the DNAs of a cohort of 30 polyendocrine patients by PCR amplification with specific primers (Sigma-Genosys Ltd., St. Louis, MO, USA) for exon 2: forward 5′-GAATCATCCTCCAAACAG-3′ and reverse 5′AGATGAGAACAATCAGTACC-3′. The amplification was carried out with Power SYBR Green PCR Master Mix (Applied Biosystems, Foster City, CA, USA), generating PCR products of 545 bp that were purified using the NucleoSpin Gel and PCR Clean-Up kit (Macherey-Nagel, Düren, NRW, Germany). Sequencing was carried out as reported above.

### 4.4. Functional Studies

#### 4.4.1. Confocal Microscopy Analysis

To compare the expression and distribution of TIM-3, Peripheral Blood Mononuclear Cells (PBMCs) isolated from the patient carrying the c.331C>T (p.Arg111Trp) and the c.302C>T (p.Thr101Ile) *TIM-3* variants and two healthy donors were quickly thawed in a 37 °C water bath, transferred to a pre-warmed culture medium and centrifuged at 1200 rpm for 5 min. The cells were then counted and washed two times with Phosphate Buffer Saline (PBS). 1.5 × 10^6^ cells per condition were fixed with 4% paraformaldehyde (PFA) at 4 °C for 10 min. A total of 100 μL of each cell suspension was placed on microscopy slides inside a PAP Pen delimited spot and dried at room temperature (RT) for 30 min. The samples were permeabilized with 0.1% Triton X-100 in PBS for 20 min at RT and blocked with 1% bovine serum albumin (BSA) and 5% normal goat serum (NGS) in PBS for 30 min at RT. The samples were then transferred to a humidity chamber; incubation with mouse α-TIM-3 monoclonal antibodies (Invitrogen, Carlsbad, CA, USA, MA5-32840) was performed for 1 h at RT (1:25 in blocking solution). Then, the samples were washed three times with PBS for 5 min each and treated with F(ab’)2-goat anti-mouse IgG (H + L) cross-adsorbed secondary antibodies (Invitrogen, Carlsbad, CA, USA, A21425) for 1 h at RT (1:500 in 1% BSA/PBS blocking solution). The samples were washed again three times with PBS, and cover slips were mounted on the microscopy slides using 60% glycerol in PBS.

Confocal microscopy was performed with a Leica TCS-SP8X laser-scanning confocal microscope (Leica Microsystems, Mannheim, Germany) equipped with a tunable white light laser (WLL) source, a 405 nm diode laser, 3 internal spectral detector channels (PMT) and 2 GaAsP internal spectral detector channels (HyD). Sequential confocal images were acquired using an HC PL APO 63× oil-immersion objective (1.40 numerical aperture, NA, Leica Microsystems) with a 1024 × 1024 format and a scan speed of 400 Hz.

The acquired images were analyzed to compare the fluorescence intensity of the TIM-3 protein in the PBMCs derived from the healthy donors and patients. Fluorescence intensity was quantified using Metamorph software (7.8.13.0). For each condition (healthy donors or patient), 115 individual cells were manually segmented to obtain the integrated fluorescence intensity associated with TIM-3 for each cell. The mean fluorescence intensity (MFI) and standard deviation (SD) were calculated for each group. Statistical analysis was performed using a *t*-test to assess the significance of the difference between the two groups.

#### 4.4.2. RT-qPCR

In order to quantitatively compare the *TIM-3* SNPs’ mRNAs, PBMCs isolated from the patient carrying the c.331C>T (p.Arg111Trp) and the c.302C>T (p.Thr101Ile) variants (vide infra) and two healthy donors as controls were quickly thawed in a 37 °C water bath, transferred to a pre-warmed culture medium and centrifuged at 1200 rpm for 5 min. The total RNA was extracted using the RNeasy Plus Mini Kit (Qiagen, Hilden, Germany) according to the manufacturer’s instructions. The quality and concentration of the extracted RNA were assessed using a NanoDrop ND-1000 spectrophotometer (Thermo Fisher Scientific, Waltham, MA, USA). For reverse transcription, the RNA was converted to cDNA using SuperScript II reverse transcriptase (Thermo Fisher Scientific) and random primers (Thermo Fisher Scientific), according to the manufacturer’s instructions, in a total reaction volume of 20 µL. Quantitative PCR was performed using the QuantStudio 7 Real-Time PCR System (Applied Biosystems, Foster City, CA, USA) and Power SYBR Green Master Mix (Applied Biosystems, Foster City, CA, USA). Each 20 µL reaction contained 10 µL of SYBR Green Master Mix, 0.15 µM of forward and reverse primers (GAPDH Fw: 5′-CGACCACTTTGTCAAGCTCA-3′, GAPDH Rev: 5′-AGGGGTCTACATGGCAACTG-3′; TIM-3 Fw: 5′-CCTGTCCTGTGTTTGAATG-3′, TIM-3 Rev: 5′-GTTTGATGACCAACTTCAGG-3′) and 9 µL of the cDNA template, corresponding to 50 ng of total cDNA.

The qPCR cycling conditions were as follows: initial activation at 50 °C for 2 min and then 95 °C for 2 min, followed by 40 cycles of denaturation at 95 °C for 15 s, annealing at 60 °C for 1 min and extension at 72 °C for 30 s. A melt curve analysis was performed to ensure the specificity of the amplification products. All reactions were run in triplicate, and no-template controls (NTCs) were included to check for contamination. The gene expression levels were represented as TIM-3/GAPDH relative expression and calculated by the ΔΔCt method.

## Figures and Tables

**Table 1:** *TIM-3* variants identified in the APS3 patient DNA. AR = Autosomal Recessive; HGVSC = Human Genome Variation Society coding sequence name; HGVSP = Human Genome Variation Society protein sequence name; HET = heterozygous.

Gene	OMIM Inheritance	HGVSC	HGVSP	Zygosity	Codon	dbSNP
*HAVCR2*	AR	c.331C>T	p.Arg111Trp	HET	Cgg/Tgg	rs145478313
*HAVCR2*	AR	c.302C>T	p.Thr101Ile	HET	aCt/aTt	rs147827860

**Table 2:** Clinical presentation, laboratory and instrumental parameters of the APS patient (altered parameters are in bold). Years = yrs; nr = normal range; nv = normal value; ALT = alanine transaminase; AST = aspartate transferase; GGT = gamma-glutamyl transferase; ALP = alkaline phosphatase; PT = prothrombin time; pos = positive.

Age	Clinical Manifestation/Therapy	Ab	Laboratory and Instrumental Parameters
1.8 yrs	Patent ductus arteriosus Type 1 diabetes mellitus (insulin treatment started) IgA deficit	GADAb, IAA pos IA2Ab neg	IgA 29mg/dL (nr 36–165)
3.5 yrs	Celiac disease (gluten-deprived diet)	EMA, TRGAb neg	
5 yrs	Amplatzer device treatment of patent ductus arteriosus		
12 yrs	Added metformin treatment		
13 yrs	Partial epileptic crisis Preclinical autoimmune thyroiditis	TPOAb pos TGAb neg	Ecography: thyroiditis Normal thyroid function EEG
13.3 yrs	Autoimmune hepatitis Treated with cyclosporine and azathioprine	LKMAb, ASMA pos ANA, ANCA, AMA, ARA, anti-SLA/LP, anti-SP00, anti-gp210, APCA, TRGAb neg	ALT 606 U/L (nv < 33) AST 408 U/L (nv < 32) GGT 119 U/L (nv < 40) ALP, bilirubin, α1-antitrypsin, ceruloplasmin, cupruria, PT nv IgG 20.8 g/L Hepatic agobiopsy: chronic hepatitis Screening *AIRE* neg Screening C1858T *PTPN22* neg
14.8 yrs	Obesity Azathioprine, colecalcipherol, metformin, insulin	Anti-LC1 Ab pos	Macrocytosis due to azathioprine
16.2 yrs		Anti-adrenal Ab, anti-SLA pos ASCA Ab neg	Neutropenia, leukopenia D hypovitaminosis

## Data Availability

The datasets presented in this article are not readily available because of the ethical consent concerns of the patients. All reported variants of the *AIRE*, *PTPN22* and *TIM-3* genes are already described in the literature. Requests to access the datasets should be directed to the corresponding author and clinicians Marco Cappa and Donatella Comparcola.

## Supplementary Materials

The following supporting information can be downloaded at: https://www.mdpi.com/article/10.3390/ijms252010994/s1.

## Nomenclature

21-OHAb, 21-hydroxylase autoantibodies; ACAs, adrenal cortex autoantibodies; AIDs, autoimmune diseases; AIRE, autoimmune regulator; AITD, autoimmune thyroid disease; ALP, alkaline phosphatase; ALT, alanine transaminase; AMAs, mitochondrial autoantibodies; ANAs, nuclear autoantibodies; ANCAs, neutrophil cytoplasmic autoantibodies; APCAs, parietal cell autoantibodies; APECED, Autoimmune–Polyendocrinopathy-Candidiasis-Ectodermal dystrophy syndrome; APS, autoimmune polyglandular syndrome; APS1, autoimmune polyglandular syndrome Type 1; APS2, autoimmune polyglandular syndrome Type 2; APS3, autoimmune polyglandular syndrome Type 3; APS3A, AITD and endocrinopathies excluding AD; APS3B, AITD with gastrointestinal, hepatic or pancreatic autoimmunity; APS3C: AITD with skin, neurological and hematological diseases; APS3D, AITD with autoimmune rheumatological, cardiac and vascular diseases; APS4, autoimmune polyglandular syndrome Type 4; AR, Autosomal Recessive; ARAs, reticulin autoantibodies; AST, aspartate transferase; β2GP-1IgGAb, beta2glycoprotein I-IgG autoantibodies; β2GP-1IgMAb: anti-beta2glycoprotein I-IgM autoantibodies; CAb, anti-cardiolipin autoantibodies/cyclic citrullinated peptide autoantibodies; Ceacam 1, carcinoembryonic antigen cell adhesion molecule 1; CMC, chronic mucocutaneous candidiasis; CTLA4, cytotoxic T lymphocyte antigen 4; DGP-IgGAb: deaminated gliadin-IgG autoantibodies; dsDNAAb, double-stranded DNA autoantibodies; DSF70Ab, dense fine speckled 70 autoantibodies; ECLIA, electrochemiluminescence immunoassay; ELISA, enzyme-linked immunosorbent assay; ENA, extractable nuclear antigen autoantibodies; FOXP3, forkhead box P3; GADAb, glutamic acid decarboxylase isoform 65 autoantibodies; Gal-9, Galectin-9; GGT: gamma-glutamyl transferase; GP210, anti-glycoprotein GP210 autoantibodies; HAVCR2, hepatitis A virus cell receptor 2; HGVSC, Human Genome Variation Society coding sequence name; HGVSP, Human Genome Variation Society protein sequence name; HLA, human leukocyte antigen; HMGB1, high-mobility group box 1; IA2Ab, tyrosine phosphatase-related islet antigen 2 autoantibody; IAAs, insulin autoantibodies; IFIAb, intrinsic factor autoantibodies; IgV, Immunoglobulin variable; IL2Ra, interleukin 2 receptor alpha; LC1Ab, liver cytosol type 1 autoantibodies; LKMAb, anti-liver kidney microsomal autoantibodies; MHC, major histocompatibility complex; MICA, MHC class I chain-related gene A; NK: natural killer; OMIM: Online Mendelian Inheritance in Man; ORPHA, Orphanet (a reference portal for information on rare diseases); PT, prothrombin time; PtdSer, phosphatidylserine; PTPN22, protein tyrosine phosphatase non-receptor type 22; RA, rheumatoid arthritis; RAb, ribosomal autoantibodies; RMSD, root mean square deviation; RMSF, root mean square fluctuation; SCL-70Ab, SCL-70 antigen autoantibodies; SLA/LPIgG, soluble liver antigen/liver pancreas autoantibodies; SLE, systemic lupus erythematosus; SMAs, smooth muscle autoantibodies; SNPs, single-nucleotide polymorphisms; SP100Ab, anti-SP100 autoantibodies; T1D, type 1 diabetes mellitus; TIM-3, T cell immunoglobulin and mucin domain 3; TgAb, thyroglobulin autoantibodies; TNFα, tumor necrosis factor alpha; TPOAb, thyroperoxidase autoantibodies; TRAb, thyrotropin-receptor autoantibodies; TRGAb, anti-transglutaminase-IgA autoantibodies; TSH, thyrotropin; VNTR, variable number of tandem repeats; WES, whole-exome sequencing; ZnT8Ab, zinc transporter 8 autoantibodies.
