# Supplementary material for: The Putative Role of TIM-3 Variants in Polyendocrine Autoimmunity: Insights from a WES Investigation
Source: Int J Mol Sci. 2024 Oct 12;25(20):10994. doi: 10.3390/ijms252010994 (PMC11506967; doi:10.3390/ijms252010994)
Supplement: Supplementary file 1 [file ijms-25-10994-s001.zip › Supplementary Figure S1 - 2nd revision.pdf]

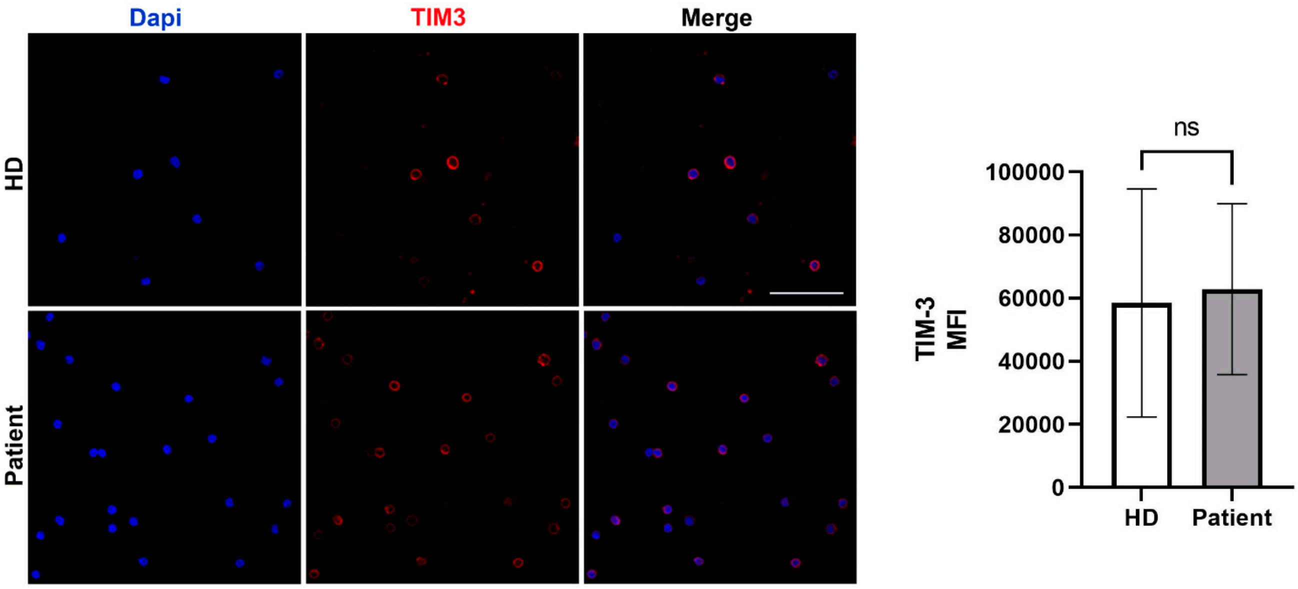

Supplementary Figure S1. Comparative LSCM analysis of Peripheral Mononuclear Blood cells (PBMCs) stained for TIM-3 protein isolated from a healthy donor and the patient. No significant differences in TIM-3 fluorescence intensity were observed between the healthy and patient-derived PBMCs. Scale bar: 50 µm.
