# Supplementary material for: The Putative Role of TIM-3 Variants in Polyendocrine Autoimmunity: Insights from a WES Investigation
Source: Int J Mol Sci. 2024 Oct 12;25(20):10994. doi: 10.3390/ijms252010994 (PMC11506967; doi:10.3390/ijms252010994)
Supplement: Supplementary file 1 [file ijms-25-10994-s001.zip › Supplementary Table S1.pdf]

Supplementary Table S1. Clinical, immunological and genetic characteristics of polyendocrine patients (3) screened for the identified *TIM-3* variants

Patient N°	Gender	Age (actual, yrs)	Age at referral	APS	Clinical manifestations	Auto Abs	TIM-3 c.331C>T c.302C>T	*AIRE* pattern	*AIRE* promoter SNP	C1858T *PTPN22*	Therapy
1	F	29.9	8.7	IIIA<br>IIIB	T1D, celiac disease, HT	**TgAb, TPOAb, GADA, IA2Ab, APCA pos**	WT	het<br>IVS9 delC +78	-230Y (C/T het)	WT	Insulin
2	F	30.1	9.2	IIIA<br>IIIC	T1D, vitiligo	**ANA, ASMA, TgAb, GADA, IA2Ab, pos**<br>AMA, CAb, TPOAb, APCA, TRGAb, Adrenal Ab, anti-21-hydroxylase Ab neg	WT	het<br>IVS9+78delC	WT	WT	Insulin aspart
3	F	24.2	2.8	IV	T1D, celiac disease	**IAA, GADA pos**<br>TgAb, TPOAb, IA2Ab, TRGAb, Adrenal Ab neg	WT	hom<br>IVS9+78delC	WT	WT	Insulin aspart<br>Insulin degludec
4	M	NA	NA	IV	T1D, celiac disease	NA	WT	het<br>IVS9 +6 G>A	WT	het	NA
5	F	29	11.2	IV	T1D, celiac disease	**GADA pos**<br>TgAb, TPOAb, IAA, IA2Ab, TRGAb neg	WT	het<br>IVS9+78delC	-230Y (C/T het)	het	none
6	F	19.6	6.1	IIIC<br>IIID	HT, alopecia universalis, onicodystrophy, allergic rhinytis, arthralgias, Raynaud phenomenon, recurrent infections in pediatric age, celiac disease	**ANA, TgAb, TPOAb, CAb, Dense Fine Speckled 70kDa (DFS70) Ab pos**<br>ASMA, ANCA, ARA, RbAb dsDNA, DNA/ENA/pRIBAb, phospholipid Ab, beta2 glycoprotein Ab, IAA, GADA, IA2Ab, LKMAb, LC1Ab, APCA, TRGA, Adrenal Ab neg	WT	het<br>IVS9+6 G>A	WT	WT	Levothyroxine (LT4)<br>Vitamin D
7	F	33.6	8.9	IIIA<br>IV	T1D, hypertension, Basedow's disease, POF	**ANA, TgAb, TPOAb, GADA, IA2Ab, APCA pos**<br>AMA, ASMA, ARA, RbAb, CAb, LKMAb, LC1Ab, IFIgG, TRGAbs, DGPIgG, Adrenal Ab neg	WT	WT	WT	WT	Insulin aspart<br>Cholecalciferol<br>methimazole<br>propanolol hydrochloride<br>Folic acid
8	F	39.1	14.8	IIIA<br>IIIB	T1D, celiac disease, HT	TgAbs, TPOAb, TRGAb neg	WT	WT	WT	het	LT4<br>Insulin
9	F	32.5	5	IIIA	T1D, HT	**TPOAb, IAA pos**	WT	WT	-380S (C/G het)	WT	Insulin aspart<br>LT4

						ANA, AMA, ASMA, ARA, RAb, CAb, TgAb, GADA, IA2Ab, LKMAb, LC1Ab APCA, TRAb, TRGAb, Adrenal Ab, anti-21-hydroxylase Ab neg			-230Y (C/T het)		
10	F	30.4	0.6	IV	T1D, celiac disease, Turner syndrome	**IAA GADA IA2Ab pos** ANA, AMA, ASMA, ARA, RAb, CAb, TgAb, TPOAb, LKMAb, LC1Ab, APCA, TRGAb, DGPIgG, Adrenal Ab neg	WT	het IVS9+78delC	WT	het	Insulin lispro<br>Insulin degludec
11	M	33.4	13.8	IIIA<br>IIIB	T1D , HT, autoimmune athrophic gastritis	**TPOAb, APCA pos** ANA, AMA, ASMA, ARA, RAb, CAb, TgAb, GADA, IA2Ab, LKMAb, LC1Ab, IFIgG, TRAb, TRGAb, Adrenal Ab neg	WT	het c.834 C>G (S278R) het IVS9+6 G>A het IVS9+78delC	-230Y (C/T het)	WT	LT4<br>Insulin aspart, Insulin degludec
12	F	30.2	6	IIIA	T1D, HT	**ASMA, TgAb, TPOAb, IAA pos** ANA, AMA, ARA RAb, CAb, GADA, IA2Ab, LKMAb, LC1Ab, IFIgG, APCA, TRGAb, Adrenal Ab, snti-21-hydroxylase Ab neg	WT	het IVS9+78delC	WT	WT	LT4<br>Insulin aspart<br>Insulin glargine
13	F	34.1	10.5	IIIA<br>IIIB<br>IIIC	T1D, HT, vitiligo, chronic gastritis	**ANA, CAb, TgAb, TPOAb, GADA IA2Ab, APCA pos** AMA, ASMA, ARA, RAb, anti-beta2 glycoprotein Ab, LKMAb, LC1Ab, IFIgG, TRGAb. Adrenal Ab neg	WT	hom IVS9+78delC	WT	WT	LT4 Insulin aspart Insulin glargine
14	M	36.9	12.6	IV	T1D, celiac disease	**TPO, IAA pos** GADA, IA2Ab, TgAb, TRGAb, anti-21-hydroxylase Ab neg	WT	het IVS9+78delC	-230Y (C/T het)	WT	Insulin aspart
15	M	38.8	16.6	IIIA	T1D, HT	**TPOAb, IAA, IA2Ab pos** ANA, AMA, ASMA, ARA, RAb, CAb, TgAb, GADA, LKMAb, LC1Ab, APCA,	WT	het c.834 C>G (S278R)	-230Y (C/T het)	WT	Human insulin Insulin detemir<br>LT4

						TRGAb,<br>Adrenal Ab neg					
16	M	26.1	5.1	IV	T1D, celiac disease	**IAA,**<br>**IA2Ab pos**<br>ANA,<br>AMA,<br>ASMA,<br>ARA,<br>RAb,<br>CAb,<br>TgAb<br>TPOAb<br>GADA<br>LKMAb,<br>LC1Ab,<br>APCA,<br>TRGAb<br>DGPIgG<br>Adrenal Ab neg	WT	het<br>IVS9+78delC	-230Y<br>(C/T het)	het	Human insulin,<br>Insulin glargine
17	M	30.2	8.3	IV	T1D, celiac disease	**IAA,**<br>**GADA,**<br>**IA2Ab,**<br>**IFIgG,**<br>**APCA pos**<br>ANA,<br>AMA,<br>ASMA,<br>ARA,<br>RAb,<br>CAb,<br>TgAb,<br>TPOAb,<br>LKMAb,<br>LC1Ab,<br>TRAb,<br>TRGAb,<br>DGPIgG,<br>Adrenal Ab, anti-21-hydroxylase Ab neg	WT	WT	-230Y<br>(C/T het)	WT	Human insulin,<br>Insulin detemir,<br>Insulin glulisine
18	M	19.6	11.4	IIIC	HT, alopecia areata	**TPOAb, IAA,**<br>**GADA pos**<br>ANA, AMA, ASMA, ARA, Rb Ab, Cab, TgA,b, IA2Ab, LKMAb, LC1Ab, TRGAb, APCA, Adrenal Ab, neg	WT	het<br>IVS9+6G>A<br>hom<br>IVS9+78delC	WT	WT	
19	M	25.5	2.8	IIIA<br>IIIB	T1D, celiac disease, HT	**TgAb, TPOAb,**<br>**IAA, GADA, IFIgG,**<br>**APCA pos** ANA, AMA, ASMA, ARA, RbAb, CAb, IA2Ab, LKMAb, LC1Ab,<br>TRGAb, DGPIgG,<br>Adrenal Ab neg	WT	het<br>IVS9 delC<br>+78	-230Y<br>(C/T het)	hom	Insulin
20	F	25.5	3.8	IV	T1D, celiac disease, vitiligo	**IAA,**<br>**GADA,**<br>**IA2Ab pos**<br>ANA,<br>AMA,<br>ASMA,<br>ARA,<br>RAb,<br>CAb,<br>Beta2 Glycoprotein Ab,<br>TgAb,<br>TPOAb,<br>LKMAb,<br>LC1Ab,<br>APCA,<br>TRGAb,	WT	het<br>IVS9 +6<br>G>A<br>het<br>IVS9 delC<br>+78	-230Y<br>(C/T het)	WT	Insulin aspart

						DGPIgG, anti-21-hydroxylase Ab neg					
21	F	26.6	4.4	IIIA IIIC	T1D, vitiligo, HT, scoliosis	**APCA, Adrenal Abs pos** TgAb, TPOAb neg	WT	WT	WT	WT	Insulin aspart Insulin degludec
22	F	22.9	8.1	II IIIB	Addison's disease, celiac disease, HT	**ANA, TgAb, TPOAb, Adrenal Ab pos** AMA, ASMA, ANCA, ARA, RAb, IAA, GADA, IA2Ab, LKMAb, LC1Ab, IFIgG, APCA, TRAb, TRGAb, DGPIgG neg	WT	WT	WT	WT	Hydrocortisone Fludrocortisone LT4
23	M	14.6	12.8	IIIB	autoimmune hepatitis, HT	ANA, ASMA, ANCA RAb, LKMAb, SLAIgG, LC1Ab neg	WT	WT	-655R (A/G het), -230Y (C/T het)	WT	Cyclosporine Azathioprine Ursodeoxycholic Acid LT4
24	M	30.2	13.5	IIIA	T1D, HT	**IAA, IA2Ab pos** ANA, AMA, ASMA, ANCA, ARA, RAb, CAb, TgAb, TPOAb, GADA, LKMAb, LC1Ab, APCA, TRGAb, Adrenal Ab, anti-21-hydroxylase Ab neg	WT	NT	WT	WT	Insulin aspart, Insulin degludec
25	M	40.6	14.2	IIIA	T1D, HT	**ANA, TPOAb, GADA, IA2Ab, pos** AMA, ASMA, ARA, RAb, ACA, TgAb, LKMAb, LC1Ab, APCA, TRGAb neg	WT	het IVS9+78delC	WT	WT	Insulin, Bisopropol fumarate
26	F	35.4	15	IIIA	T1D, HT	**TgAb, TPO Ab, IAA, GADA, IA2Ab pos** ANA, AMA, ASMA, ARA, RAb, CAb, LKMAb, LC1Ab, APCA, TRGAb, Adrenal Ab neg	WT	het c.834 C>G (S278R) het IVS9+6G>A het IVS9+78delC	WT	het	LT4, Insulin aspart, Insulin degludec
27	F	25.8	4.7	IIIA IV	T1D, HT	**TPOAb, IAA, IA2Ab, TRGAb pos** ANA, AMA, ASMA, CAb,	WT	het IVS9+78delC	WT	WT	None

						TgAb, GADA, APCA, TRA, DGPIgG, Adrenal Ab, anti-21-hydroxylase Ab neg					
28	F	22.9	0.9	IIIA	T1D, HT	**TPOAb, IAA, GADA pos** ANA, AMA, ASMA, ARA, RAb, CAb, TgAb, IA2Ab, LKMAb, LC1Ab, APCA, TRGAb, DGPIgG, Adrenal Ab, anti-21-hydroxylase Ab neg	WT	het c.834 C>G (S278R) het IVS9+78delC	WT	WT	Insulin aspart, Insulin glargine
29	M	35.7	3.9	IIIA	T1D, HT	**ANA, TgAb, TPOAb, IAA, GADA, IA2Ab pos** AMA, ASMA, ARA, RAb, anti-dsDNA Ab, CAb, LKMAb, LC1Ab, APCA, TRGAb, Adrenal Ab neg	WT	het IVS9+78delC	WT	het	Insulin aspart
30	F	18.4	3.8	IIIB IIIC	alopecia universalis, HT, celiac disease, allergic rhinitis, family history for myasthenia gravis	**TPOAb pos** ANA, AMA, ASMA, ARA ANCA, RbAb, TgAb, IAA, GADA, IA2Ab, LKMAb, LC1Ab, APCA, TRGAb, DGPIgG, , Adrenal Ab neg	WT	het IVS9 delC +78	-230Y (C/T het)	WT	None

NA= not available; HT= Hashimoto's thyroiditis; POF= primary ovarian failure
